# Overcoming Barriers in Photodynamic Therapy Harnessing Nanogenerators Strategies

**DOI:** 10.7150/ijbs.100317

**Published:** 2024-10-14

**Authors:** Yi Zhou, Pingjin Zou, Xingmin Chen, Ping Chen, Min Shi, Jinyi Lang, Meihua Chen

**Affiliations:** 1 Department of Abdominal Oncology, Radiation Oncology Key Laboratory of Sichuan Province, Sichuan Clinical Research Center for Cancer, Sichuan Cancer Hospital & Institute, Sichuan Cancer Center, Affiliated Cancer Hospital of University of Electronic Science and Technology of China, Chengdu 610041, China.; 2 Department of Radiation Oncology, Radiation Oncology Key Laboratory of Sichuan Province, Sichuan Clinical Research Center for Cancer, Sichuan Cancer Hospital & Institute, Sichuan Cancer Center, Affiliated Cancer Hospital of University of Electronic Science and Technology of China, Chengdu 610041, China.; 3 Department of Pathology, Sichuan Clinical Research Center for Cancer, Sichuan Cancer Hospital & Institute, Sichuan Cancer Center, Affiliated Cancer Hospital of University of Electronic Science and Technology of China, Chengdu 610041, China.

**Keywords:** photodynamic therapy, nanogenerators, treatment resistance, cancer treatment, reactive oxygen species

## Abstract

Photodynamic therapy (PDT) represents a targeted approach for cancer treatment that employs light and photosensitizers (PSs) to induce the generation of reactive oxygen species (ROS). However, PDT faces obstacles including insufficient PS localization, limited light penetration, and treatment resistance. A potential solution lies in nanogenerators (NGs), which function as self-powered systems capable of generating electrical energy. Recent progress in piezoelectric and triboelectric NGs showcases promising applications in cancer research and drug delivery. Integration of NGs with PDT holds the promise of enhancing treatment efficacy by ensuring sustained PS illumination, enabling direct electrical control of cancer cells, and facilitating improved drug administration. This comprehensive review aims to augment our comprehension of PDT principles, explore associated challenges, and underscore the transformative capacity of NGs in conjunction with PDT. By harnessing NG technology alongside PDT, significant advancement in cancer treatment can be realized. Herein, we present the principal findings and conclusions of this study, offering valuable insights into the integration of NGs to overcome barriers in PDT.

## Introduction

Cancer has emerged as a major cause of mortality 1. Conventional cancer therapies, encompassing chemotherapy, radiotherapy, targeted therapy, and immunotherapy, are hampered by notable drawbacks such as severe side effects and toxicity towards healthy cells 2, 3. Photodynamic therapy (PDT) emerges as an appealing alternative with enhanced efficacy, reduced side effects, and heightened precision 4, 5. PDT employs light and photosensitizers (PSs) to selectively harm cancer cells by generating reactive oxygen species (ROS) 6, 7. Nonetheless, PDT encounters hurdles like inadequate tumor targeting, restricted light penetration, diminished ROS production, and the emergence of cancer cell resistance.

To address challenges, nanogenerators (NGs) have emerged as a potential solution offering several advantages. These self-powered systems convert biomechanical energy into electrical energy 8, 9, characterized by compactness, cost-effectiveness, and high-power density output 10, 11. NGs, including Triboelectric Nanogenerators (TENGs) and Piezoelectric Nanogenerators (PENGs), have made significant strides in influencing cellular responses 11, 12, particularly in precise cancer treatment 13, 14. TENGs have demonstrated efficacy in promoting cell growth, differentiating mouse embryonic osteoblast 15, 16, and guiding nerve cell growth 17. PENGs have emerged as key players in regulating cell behavior through pathways like MAPK/ERK and cyclic adenosine monophosphate(cAMP) 18, promoting cell adhesion 19, activating downstream signaling cascades, enhancing macrophage motility 20, and inducing neurite outgrowth 21. These advancements highlight the potential of NGs in positively impacting cellular behavior in cancer therapy. By enhancing ROS production within the tumor microenvironment (TME), NGs contribute to improved therapeutic outcomes while minimizing damage to healthy tissues. Notably, an implantable biodegradable TENG nanofiber patch enhances hydroxyl radical production in specific weakly acidic TME conditions 14. Another NE based gas-therapy system, can realize nitric oxide (NO) releasing over the blood-brain barrier (BBB) to realize the precise intracranial glioblastoma treatment 13.

NGs offer innovative solutions to combat resistance in PDT by addressing key challenges through various mechanisms. They can enhance oxygen supply within the TME 22, facilitate targeted drug delivery of PSs 23, 24, amplify ROS generation 25, 26, enable synergistic combination therapies 27, 28, modulate the TME 29, 30, induce immunogenic cell death 31, 32, disrupt DNA repair mechanisms 33, influence angiogenic effects 34, 35. For instance, portable wearable NG-based devices can sustainably power PSs to directly impact cancer cells 36. NG-driven drug delivery systems address resistance mechanisms in cancer treatment, improving targeting 13, 23, 24, light penetration 37, and ROS generation 26, 38 to enhance PDT efficacy.

This review aims to provide a comprehensive overview of the fundamental principles of PDT, impediments to its effectiveness, and mechanisms of resistance. It also explores the benefits of nanotechnology-enabled (NE) approaches in augmenting PDT efficacy. The physicochemical attributes of nanomaterials and their potential applications in managing deep-seated cancers, modulating cellular apoptosis pathways, overcoming resistance to PDT, and monitoring biochemical products are thoroughly examined. By identifying strategies that can enhance the efficacy of PDT, this review contributes to the progression of cancer treatment. Finally, critical considerations for the clinical implementation of the combination of PDT and NGs in cancer treatment are discussed.

## 1. PDT and the Barriers

**Figure 1:**
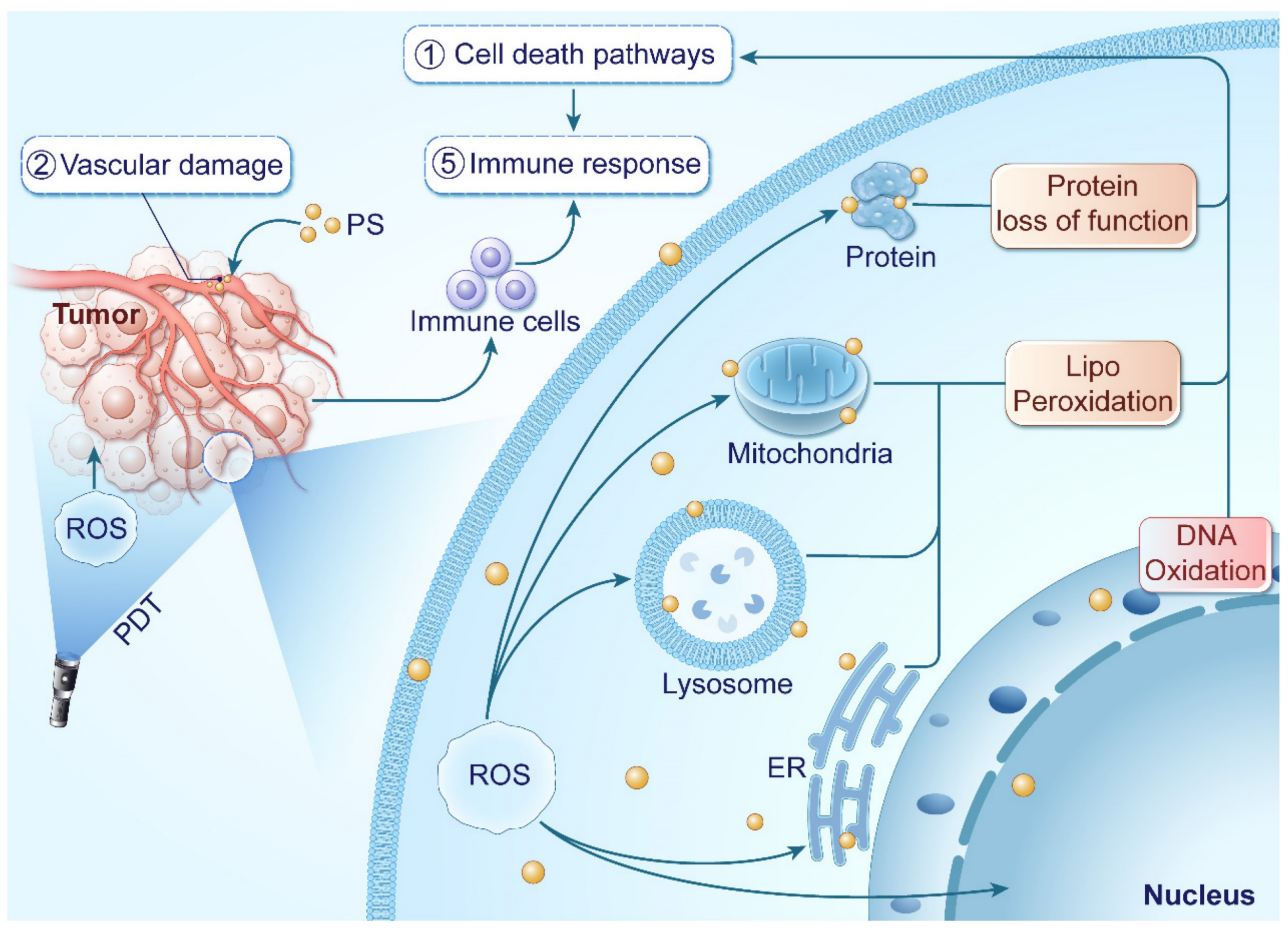
PDT leverages light exposure and PSs to generate ROS within tumor tissues, inducing tumor inhibition. ROS target cancer cells and blood vessels, disrupting cellular functions and blood supply. They also initiate an immune response against tumor metastasis.

PDT represents a promising therapeutic modality involving the introduction of PSs into tumor tissues, succeeded by light exposure at a specific wavelength. PDT initiates the production of ROS within the TME, culminating in cancer cell eradication. Within PDT, PSs convert light energy into chemical energy upon illumination in the presence of oxygen, leading to ROS generation through two photochemical processes involving adjacent molecules. The initial excited state of the PS (singlet state denoted as 1PS•) is excited to a more stable excited triplet state (3PS•), which then generates ROS, effectively eliminating tumor cells 39. In addition to tumor cell destruction, PDT can target tumor vascular endothelial cells, boost immune responses, and trigger local inflammation, collectively enhancing its therapeutic efficacy. The mechanisms underlying the anti-cancer effects of PDT are illustrated in Figure 1.

### 1.1. Dual mechanisms of Photodynamic Reactions

PDT operates through two primary mechanisms involving the generation of ROS: type I and type II reactions. Type I reaction involves complex electron transfer steps, leading to the generation of various ROS, including superoxide radicals (O_2_^-^), hydrogen peroxide (H_2_O_2_), and hydroxyl radicals (•OH). These reactions initiate upon PS photon absorption, involving energy transfer and single electron reduction processes. This process includes the singlet excited state of the PS (1PS•), where energy transfer occurs upon its return to the ground state, forming an excited triplet state (3PS•), interacting with molecular oxygen to generate singlet state PS and the excited state of singlet oxygen (^1^O_2_) that interact with cell membrane lipids 40. Initial single electron transfer steps generate PS radical anions, which react with oxygen to produce O_2_^-^. O_2_^-^then engages in redox reactions to produce H_2_O_2_, subsequently undergoing single electron reduction to form •OH. The Type II reaction transfers energy to the ground state triplet of molecular oxygen, generating the ground state singlet of the PS and the excited state of ^1^O_2_. This energy transfer elevates molecular oxygen to a higher energy state, shifting it from the ground state to an excited triplet state (^3^O_2_), while the PS reverts to its ground state. The ^3^O_2_ undergoes intersystem crossing to form ^1^O_2_, a potent cytotoxic agent pivotal in PDT 41, 42.

Oxygen concentration significantly influences PDT efficacy by modulating treatment reactions. Tumor hypoxia, characterized by low oxygen levels, can diminish PDT effectiveness, particularly in type II PDT 42-45. While type I PDT reactions can proceed independently of oxygen, its presence influences overall PDT efficacy. In oxygen-rich environments, type I reactions can generate ROS beyond ^1^O_2_, contributing to cellular damage and amplifying PDT's cytotoxic effects 43. Type I and type II processes can occur simultaneously, factors like tumor cell type, PS characteristics, oxygen levels, and tumor oxygenation complexity influence the relative occurrence of type I and type II reactions. Moreover, hypoxia impacts tumor metastasis, drug resistance 46, neovascularization 47, and the tumor immune microenvironment 48, impacting treatment effectiveness.

The outcomes of PDT are influenced by various factors, including tumor cell type and PS characteristics such as photophysical and photochemical properties 49, tissue distribution 50, absorption properties 49, 51, and subcellular localization 49, 51, 52. In various tumors, differing blood oxygen levels are observed. Non-small cell lung cancers 53, liver cancers 54, and primary/metastatic brain tumors 55 are typically well-vascularized with elevated oxygen levels 54-56. This oxygen-rich environment commonly favors Type-II reactions. Tumor oxygenation complexity is influenced by factors like depth, necrotic regions, and cancer subtypes 57. Illustrating Type I and Type II photochemical reaction of different PS is also of significant value. PSs like porphyrin, boron dipyrromethene, cyanine dyes, and aggregation-induced emission molecules have been engineered for enhanced accumulation in deep tumors and exploitation of acidic/hypoxic microenvironments in Type-II PDT reactions 58. Those PSs has shown the ability of enhancing PS accumulation in deep tumor 59, and taking advantage of acidic and hypoxia TME in the Type II PDT reaction 60-62. To counter resistance, some PSs are designed for oxygen-independent Type I PDT. NanoPcA was structured to prevent aggregation-caused quenching 63. A PS complex, coupling cyclometallated Ir (III) with coumarin, was engineered for normoxic/hypoxic conditions 64. Li *et al.* devised PSs generating superoxide radical anions for enhanced photochemical properties 65. Additionally, certain PSs are tailored for both O_2_-dependent and O_2_-independent PDT, such as Ti-based Nanoscale Metal-Organic Framework (NMOF) 66, Zr-TBB NMOF 67, heterogeneous covalent organic nanosheet 68.

ROS generated during PDT exert cytotoxic effects by attacking biomolecules like lipids, proteins, and DNA 69, 70. Plasma membrane and organelles such as mitochondria, endoplasmic reticulum (ER), Golgi apparatus, and lysosomes are particularly susceptible due to the high sensitivity of lipids to ROS 71. Photodynamic reactions induce lipid peroxidation through the production of radicals 72. ^1^O_2_ can directly react with unsaturated lipids, forming lipid peroxides, which disrupt the integrity and function of biomembranes 73. Furthermore, the generated radicals can initiate free-radical chain reactions, causing secondary modifications to proteins and polynucleotides 72, 74. DNA is crucial target of ROS in PDT, and damage to DNA can trigger apoptosis in cancer cells 75, 76. The cumulative effect of these reactions disrupts the structure and functions of organelles, ultimately resulting in cell death. Common cell death modalities in PDT include apoptosis, necrosis, and autophagy. Additionally, non-conventional forms of cell death such as necroptosis, ferroptosis, pyroptosis, parthanatos, and mitotic catastrophe may also occur 77, 78.

### 1.2. Barriers of PDT

**Figure 2:**
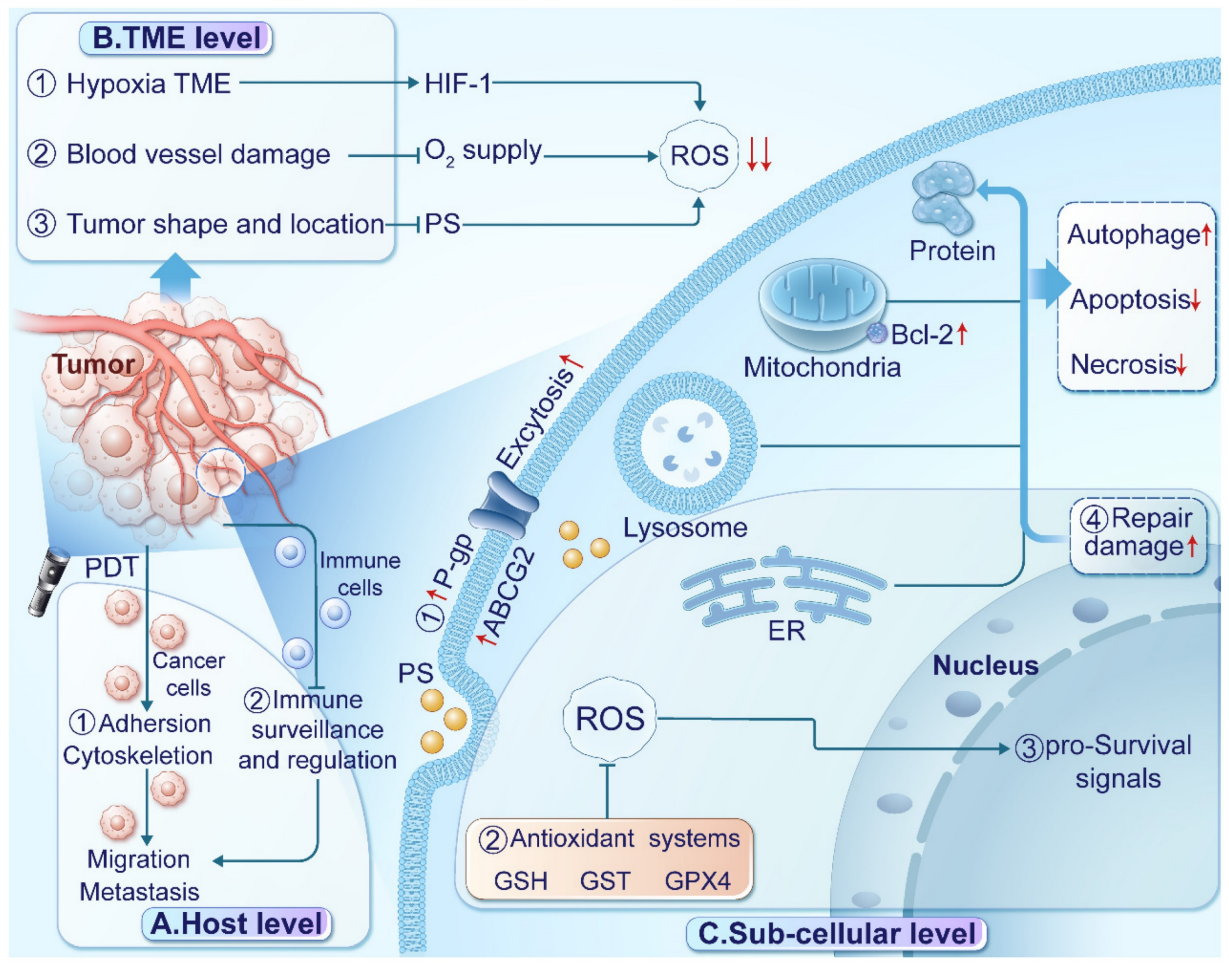
Barriers of PDT in treating cancer. (A) At the host level, ① PDT affects the cytoskeleton and cell adhesion, potentially enhancing cancer cell migration and invasion. ② Inadequate immune surveillance and regulation can limit the efficacy of PDT against distant lesions. (B) At the TME level, ① the hypoxic TME re-duces oxygen availability and stabilizes hypoxia-inducible factor 1 (HIF-1). ② Damage to blood vessels impairs oxygen supply, exacerbating hypoxia. ③ The location and shape of tumors can affect the absorption, distribution, and excitation of PSs, reducing ROS production. (C) At the subcellular level, ① Up-regulation of transport proteins excrete PSs. ② Enhanced antioxidant systems scavenge ROS in tumor tissues. ③ Activation of pro-survival signaling pathways promotes transcription and tumor progression. ④ Up-regulation of heat shock proteins facilitates the repair of proteins damaged by ROS.

PDT faces several barriers that can limit its effectiveness in treating cancer 79-81. To enhance the clinical benefits of PDT, it's essential to understand these barriers acting at the host, TME, and sub-cellular levels, which are visually represented in Figure 2.

At the host level, limitations in immune response monitoring, cancer cell migration, and treatment interruption impact PDT efficacy. Toxic effects of PSs and PDT on normal tissues also affect patient tolerance. Tumor metastasis poses a significant challenge for PDT efficacy, with limited diffusion range of ROS affecting its effectiveness on distant lesions 82. Inadequate immune response monitoring, including handling of cell debris, tumor-specific antigens, and immune activation, contributes to suboptimal efficacy against distant lesions 83. Additionally, PDT's impact on cytoskeletal dynamics, cellular plasticity, and morphology may affect cancer cell migration and invasion 84. Treatment-induced pain can lead to interruptions, diminishing overall effectiveness. Careful monitoring and managing the side effects are crucial 85, 86.

At the TME level, factors such as hypoxia, acidity, and immunosuppression contribute to resistance mechanisms against PDT. Insufficient oxygen availability and enhanced antioxidant defense mechanisms limit ROS generation, while immunosuppressive microenvironments impede immune activation. Dynamic TME changes during treatment or tumor progression play a significant role in cancer cell adaptation and resistance development 87-89. Various factors within the TME influence the mechanisms of resistance to PDT 90. For instance, hypoxia restricts ROS generation 91 by limiting oxygen availability and promotes tumor progression through upregulating hypoxia-inducible factors (HIFs) in PDT-resistant tumors 79. The acidic TME induces insufficient endogenous H2O2 and boosts cellular antioxidant defense mechanisms, restricting PDT efficacy 92. Tumor cells establish an immunosuppressive microenvironment by releasing immunosuppressive cytokines, hindering immune activation by PDT 93. Furthermore, nitric oxide (NO) generated through inducible nitric oxide synthase (iNOS/NOS2) has been associated with PDT resistance, impairing therapeutic efficacy 94. These microenvironmental factors contribute to resistance development and impact PDT outcomes.

Apart from TME level, tissue penetration depth of light is critical for PDT 95, 96. Conventional PSs are typically activated by short-wavelength ultraviolet-visible (UV-Vis) light, limiting their effectiveness in treating deep-seated tumors due to insufficient tissue penetration 97. To address this, various approaches, including near-infrared (NIR) light, X-ray radiation, and self-luminescence have been explored 98. NIR light (700-1100 nm) penetrates biological tissues more effectively than UV-Vis light (400-700 nm), enhancing treatment depth and reducing absorption by proteins and lipids 99, 100, thereby increasing light availability for tumor-targeted PSs 101, 102. By utilizing NIR light, researchers can surmount tissue penetration limitations, enhancing PDT outcomes in clinical settings 103-105. Despite its advantages, the application of NIR light in PDT presents notable challenges. Variability in tissue composition can affect light penetration and absorption, leading to inconsistent therapeutic outcomes. Furthermore, the efficacy of NIR-mediated PDT is contingent upon oxygen availability in the TME, complicating treatment in hypoxic tumors. Thermal effects from NIR exposure can damage surrounding healthy tissues if not carefully managed, and determining the optimal light dosage to mitigate unnecessary damage remains complex 106. Regulatory and safety concerns regarding NIR use must also be addressed in clinical applications. While NIR light offers deeper penetration than visible light, depth limitations persist that may affect treatment efficacy^106^. Ongoing research aims to overcome these challenges in NIR-induced PDT for Types I and II treatments 107, 108.

The suboptimal tumor-targeting capacity of PSs, leading to inefficient cancer cell uptake, presents a material performance limitation in PDT 33, 109. In clinical settings, inadequate PS localization hinders treatment efficacy by promoting PS accumulation in healthy tissues rather than specifically within tumors 110, 111. This nonspecific distribution diminishes cytotoxic effects on cancer cells and elevates the risk of harming adjacent healthy tissues 112, 113. Furthermore, variable PS concentrations within different tumor regions due to imprecise distribution impede consistent ROS generation upon light activation, thereby compromising treatment efficacy. Resolving the challenge of insufficient tumor-targeting by PSs is pivotal for enhancing PDT precision and effectiveness. Strategies like targeted delivery systems 114, nanocarriers 115, and molecular targeting agents 116 can enhance PS specificity towards cancer cells, optimizing treatment outcomes and reducing off-target effects. Overcoming this limitation can improve PDT's utility as a targeted and minimally invasive therapeutic approach across diverse cancer types.

Sub-cellular factors, including transport proteins, hypoxia-inducible factors, antioxidant systems, and pro-survival signaling pathways, play pivotal roles in PDT resistance. Transport proteins, such as P-glycoprotein (P-gp), multidrug resistance protein (MDR), and ATP-binding cassette transporter G2 (ABCG2) facilitate PS excretion from tumor cells, diminishing their retention 117, 118. The hypoxic TME influences ROS generation, activating HIF and pro-survival pathways that aid tumor progression 44, 119. Upregulated antioxidant systems like glutathione (GSH), glutathione S-transferase (GST), and glutathione peroxidase 4 (GPx4) scavenge ROS in tumor tissues 120, 121. Heat shock proteins assist in ROS-damaged protein repair 122, 123. Additionally, PDT-treated glioblastoma cells activate the AKT/mTOR signaling pathway via NF-κB signaling, regulating DNA transcription, cytokine production, and cell survival 124. Autophagy, promoting cell survival at low PDT doses by recycling damaged organelles, contributes to resistance. Negative feedback regulation decreases proapoptotic caspase-9 activity, suppressing cell death 125. The AMP-activated kinase (AMPK) pathway, activated by 5-aminolevulinic acid (5-ALA) and photoinduced stress in PDT-treated cancers, triggers autophagy, leading to tumor resistance to 5-ALA-PDT 125. Given these diverse resistance mechanisms, developing novel therapeutic strategies, such as NGs, is crucial for enhancing PDT's clinical utility and overcoming resistance challenges.

## 2. NGs

The concept of NGs originated from Zhong Lin Wang's theoretical exploration of Maxwell's equations 126. NGs convert mechanical energy into electrical energy, enabling the analysis of mechanical inputs through electrical output signals 127, 128. Unlike conventional technologies, NGs are self-sustainability and do not require external power sources.

### 2.1. Types of NGs

One type of NG is the Piezoelectric Nanogenerator (PENG), which operates based on the piezoelectric effect. Comprising an insulating piezoelectric material with electrodes on its surfaces, the PENG generates electric dipoles when strained, enhancing material polarization. This polarization establishes an electrostatic potential, balanced by electron flow through an external load. Wang's group pioneered the design of PENG in 2006 8, sparking efforts by researchers to enhance the efficiency, flexibility, and biocompatibility of PENGs for diverse applications 129, 130. PENGs efficiently convert mechanical energy into electrical energy, providing a reliable power source for PDT devices without external power. Typically made from biocompatible materials, PENGs are suitable for integration into implantable or wearable PDT systems. They can harvest energy from bodily movements, ensuring continuous power generation for extended PDT treatments. Designed at the nanoscale, PENGs enable the development of compact and minimally invasive PDT devices, offering a sustainable energy solution for prolonged use. However, PENGs may have lower power output compared to other energy harvesting technologies, potentially limiting the functionality of high-power PDT devices. They require consistent mechanical vibrations to generate electricity, which can constrain their effectiveness in static environments. The fabrication of PENGs can be complex and may necessitate specialized expertise, increasing production costs. Additionally, PENGs are sensitive to environmental factors such as temperature and humidity, which can affect performance. Integrating PENGs into existing PDT systems may also present challenges related to compatibility and optimization. Extensive research has been conducted to enhance PENG energy through material selection 131-138 and innovative device structures 135, 139, 140.

The Triboelectric Nanogenerator (TENG) is another form of nanogenerator that exploits the triboelectric effect, which arises when two materials contact each other, leading to charge transfer and subsequent electrostatic induction. Comprising two electrodes and at least one pair of triboelectric layers, the TENG generates a potential difference from an electron imbalance when the materials separate, driving electron flow between the top and bottom electrodes on the material surfaces 141. TENGs achieve high power densities, generating sufficient energy for PDT devices with varying requirements. Their output voltage surpasses that of PENGs, leading to increased research developments in this area 142. With advancements in nanomaterials, TENGs have reached an area power density of 1200 W/m² and energy conversion efficiencies of 50-85% 143. They can be scaled to meet the specific power needs of PDT devices, allowing for customization and optimization. TENGs are characterized by low cost, versatility, environmental friendliness, and high conversion efficiency. They can be designed as fibers or integrated into fabrics, making them breathable, comfortable, and suitable for low-cost, large-scale production 144. However, the size of TENGs may limit their integration into small-scale PDT systems, posing challenges for miniaturization. Furthermore, their design can be complex, necessitating careful optimization to ensure efficient energy generation in PDT applications.

### 2.2. NG-enhanced PDT

**Figure 3:**
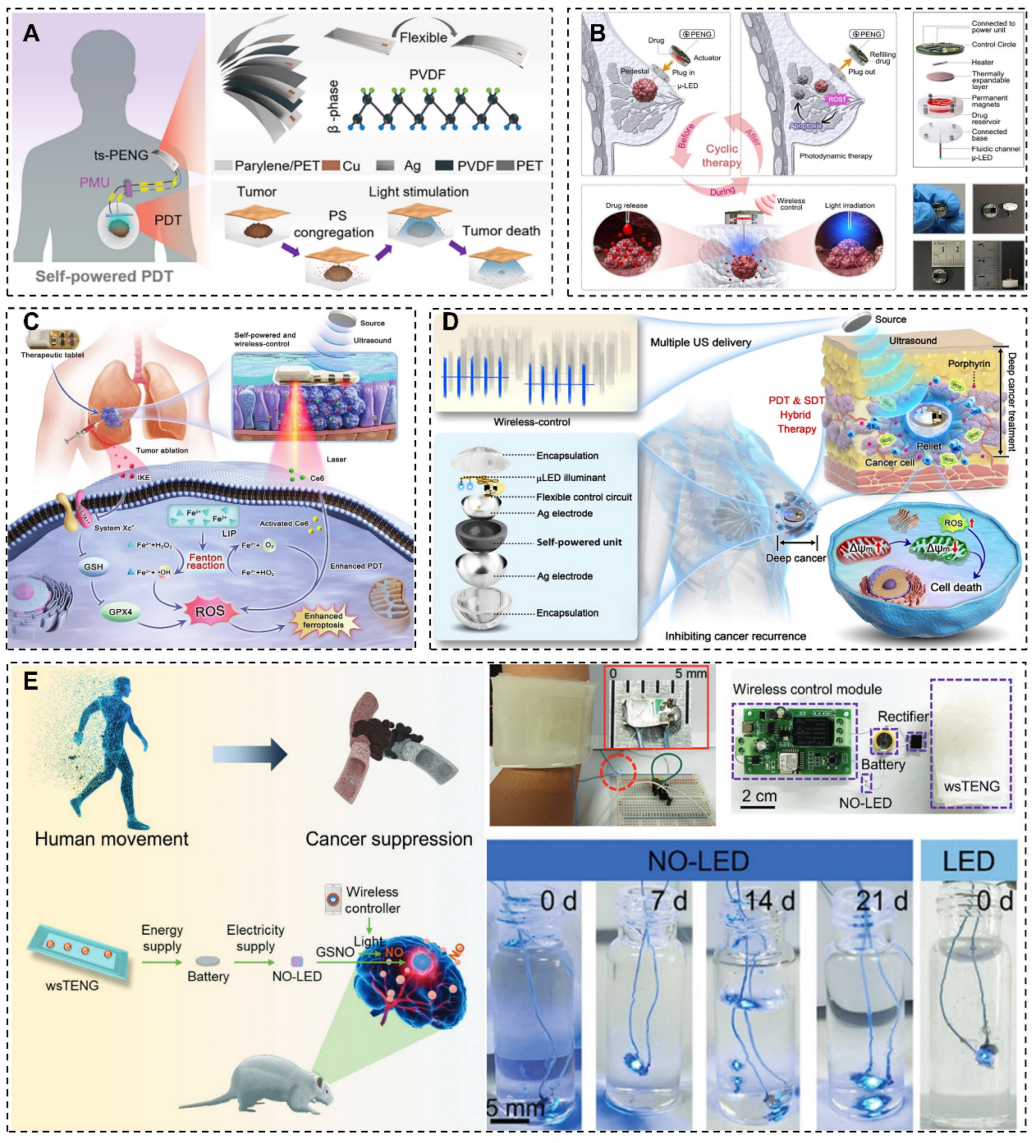
Examples of NGs and PDT combined applications. (A) Human motion driven self-powered photodynamic system for long-term autonomous cancer therapy 36. (B) A self-powered wireless detachable drug/light injector for metronomic photodynamic therapy in cancer treatment 146. (C) Implanted, wireless, self-powered photodynamic therapeutic tablet synergizes with ferroptosis inducer for effective cancer treatment 147. (D) Implantable self-powered therapeutic pellet for wireless photodynamic/sonodynamic hybrid therapy of cancer recurrence inhibition and tumor regression 148. (E) Self-powered, implantable, and wirelessly controlled NO generation system for intracranial neuroglioma therapy 13. (A) Reprinted with permission from 36. Copyright 2020 American Chemical Society. (B) Reprinted with permission, LN: 5882360170575. (D) Reprinted with permission, LN: 5882810859864. (E) Reprinted with permission, LN: 5882811413873.

The integration of NGs with PDT holds immense promise in cancer management, warranting further exploration. Previous investigations have shown encouraging results, as illustrated in Figure 3. The efficacy of NGs has been demonstrated in providing precise and sustained light sources to stimulate PSs 36, amplifying ROS generation and incident photo-electron conversion efficiency in wound healing 145, supporting detachable drug/light injector to conforms metronomic PDT at low-dose of light or drug 146, integrating ferroptosis inducer (FIN) and imidazole ketone erastin (IKE) to augment the PDT 147, and delivering therapeutic light doses into deep tissues for PDT 147.

Notably, Liu *et al.* introduced a groundbreaking self-powered PDT methodology, termed s-PDT, leveraging biomechanical energy for electricity production 36. This novel technique allows for accurate LED activation and PS stimulation to eradicate cancer cells. The distinctive attributes of the PENG position it as a prime contender for wearable medical equipment in oncology centered around PDT, offering convenient and auspicious treatment avenues.

In a recent review, the utilization of NGs in wound healing technology was explored 149. For instance, Yu *et al.* developed a TiO_2_/BTO/Au multilayered coaxial heterostructured nanorod array, demonstrating enhanced ROS generation and incident photo-electron conversion efficiency under UV-Vis light 145. This innovation holds promise for boosting ROS and •OH production, potentially enhancing PDT efficacy. The ability of the nanorod array to enhance ROS and •OH production within the UV-Vis light spectrum can be particularly advantageous in targeting and destroying cancer cells during PDT, potentially improving cancer treatment outcomes.

Chen's team presents a self-powered, wireless, and detachable drug/light injector for metronomic PDT in cancer treatment 146. The device was consistent of a micro light emitting diode (µ-LED) as light source, a syringe needle, a drug reservoir, a thermally-driven pump, and a wireless control circuit. By harnessing body motion energy through connection to various piezoelectric nanogenerators, the device can wirelessly administer precise doses of drugs and light to the subcutaneous tumor. Implementation of this device in metronomic PDT demonstrated notable efficacy, with a 57.4% reduction in average tumor volume compared to the control group. Its compact design, adaptable delivery mechanisms for drugs and light, and minimized adverse effects underscore its advantages.

Furthermore, Zou *et al.* integrates a ferroptosis inducer and imidazole ketone erastin to develop a self-powered photodynamic therapeutic tablet 147. In this device, the Fenton reaction was induced to supplement oxygen to enhance reactive oxygen species (ROS) production to augment the sensitivity of photodynamic therapy (PDT). Additionally, PDT facilitates iron ions release from the labile iron pool (LIP), and then, accelerate lipid peroxidation to inducing ferroptosis. This device demonstrated more than 85% tumor inhibition rate *in vitro* and *in vivo* experiments, due to overcome inadequate penetration and tumor hypoxia associated with PDT. Moreover, it can reduce the medication dosage to minimize adverse effects.

Additionally, a therapeutic pellet that provides wireless PDT/SDT hybrid therapy was designed by Xue's group, which contains an integrated self-powered unit, light-emitting diode illuminant, and control circuit 148. The system is powered by ultrasound energy via the piezoelectric effect to support LED, alongside direct SDT treatment, the pellet delivers therapeutic doses of light to support PDT. The implantable system efficiently treats breast cancer in mice, showed great potential to overcome the barrier of PDT in deep tumors.

These investigations spotlight the transformative potential of NGs in PDT. By surmounting the constraints of external power supplies and offering real-time monitoring, NGs position themselves as game-changers in the PDT landscape. Continued research and development are necessary to expedite the clinical adoption of NG-enhanced PDT as a breakthrough approach for various medical challenges.

**Figure 4:**
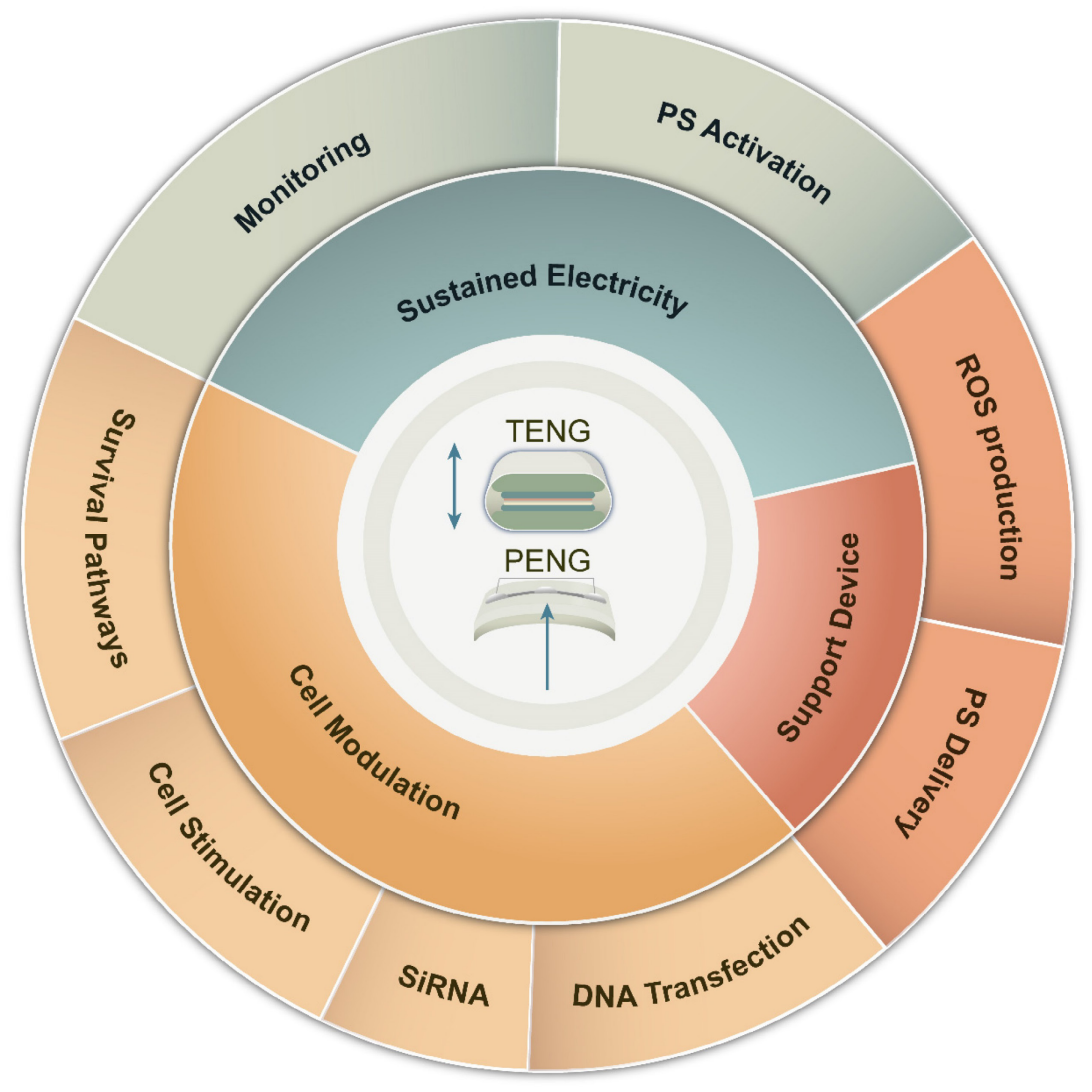
Advantages of NGs in overcoming PDT barriers. Their wearable nature provides sustained electrical power for continuous monitoring and reliable PS excitation. NGs can directly influence cancer cells through modulation of pro-survival pathways, increased adhesion, and gene therapy applications. Moreover, NGs support the development of devices that enhance PDT efficacy, enabling precise and targeted treatment delivery. These advantages highlight the potential of NGs in revolutionizing PDT and improving treatment outcomes for various medical conditions.

The intricate design of PENG/TENG brings forth additional benefits, augmenting the effectiveness of PDT in surmounting therapeutic hurdles. Therefore, a thorough exploration of the amalgamation of nanomaterial engineering and PDT is crucial to transform cancer treatment. The fusion of NGs with PDT emerges as a promising tactic to tackle the constraints linked with traditional PDT, as depicted in Figure 4. This fusion confers a distinct edge in countering PDT resistance. Serving as self-sustaining energy reservoirs, NGs expand the scope of PDT to distant or hard-to-reach regions, ensuring treatment availability. Furthermore, they furnish real-time data and responses to mechanical stimuli throughout procedures, enhancing treatment precision and efficacy.

## 3. Overcoming Tumor Resistance to PDT at the Host Level

### 3.1. NGs for Biomaterial Sensing

Advancements in biosensor technologies have transformed their power supply, capabilities, and application scenarios 150. These devices can now detect diverse biomaterial-related signals with heightened precision 151. Monitoring biomaterials involved in PDT provides valuable insights into the process, status, and efficacy of PDT, leading to enhanced effectiveness and the ability to overcome resistance.

One versatile application of NGs is their use as physical and chemical sensors. Wearable sensors, powered by NGs, gauge biochemical parameters in bodily fluids, illuminating the photodynamic process and alleviating treatment resistance 37, 152, 153. NGs such as TENGs provide high energy conversion efficiency, resilience in demanding environments, and stable adjustable outputs, rendering them ideal power sources for biomedical detection of analytes like dopamine, glucose, lactate, enzymes, various microbes, as well as chemical substances.

Peng *et al.* developed a flexible self-powered metal-semiconductor-metal (MSM) photoswitch for UV light detection, featuring a flexible gallium nitride (GaN) membrane on a polyethylene terephthalate (PET) substrate 37. The asymmetric MSM design effectively separated electron-hole pairs via the intrinsic electric field, eliminating the need for external power sources. This GaN-based photoswitch exhibited superior UV photoresponse, functioning autonomously. The piezoelectric polarization field in GaN membrane has excellent mechanical flexibility that the depletion region can be obtained to further enhance UV on/off ratio up to 154% only under 1% strain. While its attributes suggest promise for enhancing PDT precision in clinical settings, further research is needed to evaluate its applicability in deep-seated tumor monitoring.

Accurate measurement of dopamine levels holds potential as an anti-angiogenic agent in combination treatments for breast and colon cancer, thereby enhancing the efficacy of anticancer drugs 154. Jie *et al.* introduced an integrated device that incorporates a sensor leveraging the triboelectric effect for detecting neural signals and neurotransmitters 34. The device switches from Schottky to ohmic contact when exposed to a voltage pulse from the TENG. In this TENG based integrated device, shows High selectivity and sensitivity (detection limit of 0.5 μM, a linear range from 10 μM to 1 mM). The output voltage and current of the developed TENS can reach 116 V and 33 μA. The Schottky contact biosensor exhibited a tenfold higher response to dopamine compared to the ohmic contact biosensor. The speed of dopamine polymerization in extremely low concentration is the sensing limitation, further low concentration detection can be achieved by microminiaturization of TENG. The Schottky contact biosensor exhibited a tenfold higher response to dopamine compared to the ohmic contact biosensor. The device was tested for simultaneous detection of dopamine and nerve signals pre- and post-treatment. The integration of this device with the TENG shows potential for dopamine determination in cancer therapy, contributing to targeted treatments and enhancing the effectiveness of anticancer drugs.

A TENG-based preconcentrator integrated with a smartphone was developed for immune sensing applications 155. In this setup, the TENG served as a power source to drive a nanofluidic preconcentrator for electrokinetic trapping of biomolecules. The TENG operated at 3.7 Hz and a rotary TENG was used to investigate higher frequencies (7-37 Hz). The research showcased captured images of immunobeads-filled fluidic channels on a smartphone. During PDT, tumor tissue destruction can release substances like inflammatory cytokines and activate complement 156, 157. The TENG's role as a power source for the nanofluidic preconcentrator enables the electrokinetic trapping of biomolecules, facilitating immune sensing. This technology offers insights into the immune status of tumors, aiding in decisions regarding the combination of PDT with immune adjuvants. Such a combination has the potential to augment the body's immune system efficacy in combating tumors and potentially induce an "abscopal effect," bolstering the body's anti-tumor capabilities.

Glucose acts as a critical metabolic regulator influencing cancer cell sensitivity to PDT. Modulating glucose metabolism can enhance PDT efficacy by sensitizing tumors or promote resistance mechanisms, highlighting the need to target these pathways for improved outcomes. Combining glucose-modulating agents with PDT offers a promising synergistic cancer treatment strategy. For instance, 2-deoxy-D-glucose (2-DG) can alter glucose utilization pathways, amplifying PDT effectiveness and enhancing therapeutic responses 158. Glucose oxidase (GOx) significantly reshapes the TME by inducing glucose deprivation 159-161, sensitizing cancer cells to PDT. Utilizing GOx represents an innovative approach to optimizing PDT outcomes. Glucose levels can serve as biomarkers for assessing tumor responses to PDT, facilitating personalized therapeutic interventions and improving patient outcomes. TENGs have been employed for glucose sensors. One approach involves harnessing TENG-generated energy stored in a battery to power a glucose biosensor 162. Another approach uses a whirligig-inspired TENG to power commercial glucose sensors 163. This TENG harvest body motion energy from fabricated between a patterned polydimethylsiloxane (PDMS) film and an Al foil was fabricated. The maximum output of voltage and current density are up to 17 V and 0.02 μA/cm^2^, respectively. The TENG can directly illuminate 30 light-emitting diodes (LEDs) and charge a lithium-ion battery to power a glucose biosensor.

### 3.2. NGs for Metastasis Detection and Privation

Metastasis and recurrence represent significant hurdles in cancer treatment, often resulting in cancer-related deaths. is an efficacious local anti-cancer treatment, its effectiveness can be compromised by metastatic spread. In response to this challenge, researchers have devised devices incorporating piezoelectric BaTiO3 nanoparticles 19. These nanoparticles have the capability to convert ultrasonic energy into an electrical signal, facilitating cancer cell adhesion and prompting stem cell differentiation. Moreover, the surface nanotopography of biomaterials has exhibited potential in improving cell adhesion and controlling cell behavior via mechanical transduction pathways. Integrating a PENG device can potentially enhance cancer cell adhesion and mitigate metastasis. By leveraging these advancements in nanotechnology and materials science, researchers aim to address the complex issues of cancer metastasis and recurrence, ultimately advancing the landscape of cancer treatment strategies.

The identification of circulating tumor cells (CTCs) is critical in PDT. While PDT is an effective local anti-cancer treatment, its efficacy may be compromised by metastatic tumors 98. Immune responses elicited by PDT often lack the potency to eradicate metastatic growths 164, particularly due to the immunosuppressive nature of the TME 93. Early detection of CTCs is crucial for prognostic assessment and improving survival rates among cancer patients with metastasis. CTCs act as valuable biomarkers for evaluating prognosis across various carcinoma types 165. However, current *ex vivo* CTC isolation technologies capture only a small number of CTCs due to the restricted blood volumes obtained from single venipunctures, resulting in statistical variability and an inaccurate representation of tumor cell heterogeneity. To overcome these challenges, Daniel F. Hayes and his team have developed an innovative *in vivo* intravascular aphaeretic CTC isolation system 166. This technology enables continuous collection of CTCs directly from a peripheral vein, allowing for the analysis of a greater number of CTCs. Such advancements facilitate precise monitoring and management of tumor metastasis by providing more comprehensive and representative data. Furthermore, NGs have shown potential in enhancing cancer cell adhesion, which may improve CTC analysis 19, 165. This enhanced understanding of CTC dynamics can inform strategies to combine PDT with immunotherapy or other modalities, thereby strengthening anti-tumor immune responses and preventing metastasis.

## 4. Overcoming Resistance to PDT at the TME Level

### 4.1. NGs for PS Excitation

The excitation of PSs in PDT is crucial for its clinical application. Several factors such as light sources, photodynamic penetration, PS absorption, distribution, and activation influence this excitation process. Achieving effective PS activation is essential for successful PDT in clinical settings. However, limited light penetration in deep-seated tumors presents a challenge.

To address this challenge, various strategies have been explored, including the use of light with better tissue penetration and sustained light illumination. Long-wavelength light, particularly within the first (650-950 nm) and second (1000-1350 nm) NIR bio-windows, has been shown to enhance tissue penetration 167. involving photon upconversion, two-photon excitation, X-ray excitation, or internal self-luminescence of PSs have been developed to treat tumors in deep tissues 105, 168-170. One such approach is the use of a wearable twinning structure piezoelectric nanogenerator (ts-PENG) in a system called s-PDT 36. This system converts biomechanical energy into electrical energy to power a miniaturized LED (m-LED) via a power management unit (PMU), delivering light stimulation over tumor tissues. *In vivo* experiments have demonstrated that the s-PDT system effectively inhibits tumors with intermittent or continuous light stimulation.

Additionally, a TENG-based approach incorporates a nanoprodrug that combines piezoelectric nanomaterials and amphiphilic prodrug molecules 171. Ultrasound-triggered activation releases chemotherapy drugs and nitric oxide, inhibiting chemoresistance and enhancing antitumor efficiency through controlled drug and NO release.

Efficient and targeted intracellular delivery of PS biomacromolecules remains a challenge in PDT. TENGs offer a promising solution to improve PS delivery. A device combining microneedles and a TENG has been developed, showing increased drug delivery rates 172. A self-powered microfluidic transport system combining electrowetting and a freestanding mode TENG allows for precise positioning and fast response speeds in PS delivery 173.

Tumor morphology can significantly influence the efficacy of PDT. Some tumor regions may be challenging to access directly with light irradiation, leading to reduced treatment effectiveness. Conversely, flat tumors with regular shapes can receive more comprehensive light exposure, resulting in better treatment outcomes. An innovative drug delivery system based on an integrated triboelectric nanogenerator (iTENG) has been developed to address these challenges 23. This system can adapt to various implantation sites and enhance drug release during electrical field (EF) stimulation. In both *in vitro* and *in vivo* studies, the iTENG has demonstrated remarkable cytotoxicity against cancer cells.

Furthermore, a TENG-powered electrochemotherapy system featuring microneedle electrodes has shown promising results for three dimensional (3D) cells or tissues, improving therapeutic effects in irregularly shaped tumors 24. These devices are particularly well-suited for 3D tissues, aiding in the endocytosis of PSs and enhancing their penetration, absorption, and distribution within tumors. Consequently, lower PS dosages can be utilized, reducing toxicity to normal tissues. The diverse shapes of iTENGs allow for implantation in deep-seated or irregularly shaped tumors, enhancing their potential for effective treatment across various clinical scenarios. These advancements in nanogenerator technology and drug delivery systems hold promise for improving PDT outcomes in tumors with varied morphologies and complexities.

### 4.2. NGs for Monitoring the TME

Tumor tissues often exhibit an acidic extracellular environment (pH 6.5-6.9) attributed to the Warburg effect, which is linked to increased metabolic activity, rapid cell proliferation, and inadequate tissue perfusion 174. The acidic conditions in the TME can significantly influence drug transport and behavior, leading to alterations in drug characteristics. Research indicates that enhancing the pH gradient between tumor and normal tissues can improve the concentration gradient of photosensitizing drugs, thereby enhancing their lipophilicity and cellular uptake 175, 176. Alluri and colleagues have developed a self-powered biosensor for theranostics that specifically measures blood pH 177. They utilized the ionotropic gelation technique to create multifunctional biopolymer-piezoelectric composite worm structures featuring wavy and linear patterns. By adjusting the length and weight ratio of piezoelectric nanoparticles, the peak-peak voltage and current decreases around 87% and 71% for composite wavy pattern worm(CWPW) devices when the CWPWs length decreased to 56.4% (L = 1.95 to 0.85 cm) respectively. The pH-dependent conductivity of the composite linear worms (CLWs) was evaluated for potential clinical monitoring applications. The self-powered biosensor generated piezoelectric potential, serving as an independent power source to operate the CLW sensor effectively under varying pH conditions. This innovative technology demonstrates significant promise for monitoring pH fluctuations within the TME and optimizing drug delivery strategies in theranostics. By enabling real-time pH monitoring, this biosensor could potentially enhance our understanding of the TME dynamics and improve the precision of drug delivery in cancer theranostics.

Reflecting the hypoxic TME, lactate serves as a metabolic marker that helps predict treatment success and tumor behavior 178, 179. Hypoxia-induced lactate accumulation creates a resistant environment, limiting treatment effectiveness and potentially inducing lactic acidosis 180-183. Detecting lactate levels can predict PDT success, guiding treatment strategies and patient responses. TENGs have demonstrated potential for lactate detection, as well as electrochemical synthesis of nanoparticles. For instance, a self-powered system was developed to detect lactate in sweat, achieving high selectivity for lactate over interfering species like creatinine, uric acid, and glucose 184. The maximum output voltage and current density reaching 500 V and 14 mA/m^2^, respectively. Continued advancements in TENG technology could lead to the development of more efficient and convenient lactate monitoring devices, further enhancing the ability to predict PDT success, guide treatment strategies, and improve patient outcomes.

### 4.3. NGs for Modulating the TME

The hypoxic microenvironment commonly observed in tumors undergoing Photodynamic Therapy (PDT) arises from various factors such as imbalances in oxygen consumption, inefficient oxygen supply due to abnormal tumor vasculature, and the effects of conventional type II PDT 89. Rapid oxygen depletion and limited blood supply during PDT treatment can promote the development of aggressive malignant cells and treatment-resistant cell populations. Mitochondrial oxidative phosphorylation (OXPHOS) contributes to both tumor hypoxia and mitochondrial ROS production 185-187. The superoxide dismutase (SOD) catalytic cascade converts mitochondrial superoxide anions (O_2_•-) into hydroxyl radicals (OH•), enhancing Type I and Type II PDT efficacy and generating O_2_ to support oxygen-dependent Type II PDT 188-190. The interplay between OXPHOS-mediated O_2_ consumption and PDT-induced O_2_ depletion exacerbates tumor hypoxia.

Besides oxygen exhausting, ROS generated during PDT can directly harm endothelial cells, leading to a reduction in blood supply, which further aggravates hypoxia within the TME 42. Hypoxia influences the tumor immune microenvironment post-PDT, promoting angiogenic signals, cell survival, proliferation, invasiveness, and metastasis 79, 191. It also induces the transformation of macrophages into the M2 type, recruiting myeloid-derived suppressor cells (MDSCs) and inhibiting T cell activation 192, 193. To address the challenges, various strategies have been proposed. These include increasing oxygen supply, utilizing oxygen-independent PDT reactions, stimulating ROS production, and combining PDT with hypoxia-activated or oxygen-independent therapies 98, 118. By implementing these strategies, PDT can be optimized for effectiveness in hypoxic microenvironments, potentially leading to improved treatment outcomes for cancer patients.

PENG/TENG technologies can power implantable devices that actively release oxygen in hypoxic regions. For instance, a PENG-powered oxygen release system could respond to local oxygen levels, ensuring a steady supply in areas with limited oxygen availability 38. TENG/PENG technologies were used to power drug delivery systems that can trigger the release of oxygen-sensitizing agents in hypoxic tumor regions 194. This approach can enhance the efficacy of treatments like PDT by improving oxygen levels within the tumor. PENG/TENG devices were incorporated within tissue-engineered constructs to promote oxygenation in hypoxic environments 195. These sensors could trigger responses such as oxygen release or drug delivery based on the detected oxygen concentrations, enabling precise and targeted interventions. Designing self-powered oxygen generation systems based on PENG/TENG technologies that can extract oxygen from ambient sources and deliver it to hypoxic tissues 196. These systems can operate independently of external power sources, making them suitable for sustained oxygen delivery in challenging environments. The use of PENG/TENG-powered systems has also been explored in combination with other therapies to address hypoxia, for example, coupling TENG-powered oxygen release with traditional chemotherapeutic agents to improve drug efficacy in regions with low oxygen levels 25.

NGs boost ROS production in PDT through external stimulation. For example, Yao *et al.* devised a self-driven catalysis-promoting system called TENG-CatSystem 38. It comprises a self-driven TENG for electric impulses, a nanozyme featuring a 1D ferriporphyrin covalent organic framework on a carbon nanotube (COF-CNT) to generate ROS, and a conductive hydrogel embedded with COF-CNT for injection into tumor tissues. TENG-CatSystem shows promise in boosting PDT efficacy by addressing key limitations. The COF-CNT-embedded conductive hydrogel, when introduced at the tumor site, elevates local ROS levels, reduces tissue impedance, and enhances PDT precision while minimizing off-target effects. The TENG support amplifies the COF-CNT's peroxidase-like function by up to fourfold, shifting cellular responses from autophagy to apoptosis to counter PDT resistance. Additionally, the TENG-CatSystem enhances peroxidase catalytic conductivity, enabling oxygen-independent ROS generation, a potent strategy against hypoxia in PDT. The self-driven TENG ensures sustained therapeutic action, suitable for at-home use. Similarly, TENGs can boost ROS production in hypoxic TME by converting mechanical energy from body movements or blood flow 26. By leveraging NGs, researchers aim to surpass oxygen constraints and treatment resistance in hypoxic tumors, ultimately improving therapeutic outcomes.

The acidic TME with a pH of 5.6 to 6.8 originates from cancer cell hypoxia and increased glycolysis 197, 198. This acidity significantly impacts PDT efficacy through mechanisms such as PS activation, ROS generation, and treatment resistance, with implications for cancer invasion and metastasis 199, 200. Research indicates that a self-powered TENG system enhances •OH production under acidic conditions, showing promise for boosting PDT effectiveness 14. Implantable patch incorporating catalytic g-C3N4 nanosheets and the drug doxorubicin (DOX) provides electrical stimulation, significantly increasing •OH production and enhancing cancer treatment outcomes. This approach demonstrates superior tumor suppression in a breast cancer model and highlights the potential of targeting the acidic microenvironment to enhance PDT efficacy and explore combination therapies.

Improving oxygen supply in hypoxic tumors through blood flow can enhance PDT efficacy. Photothermal-mediated heating is a method that can elevate tumor blood flow and oxygen levels, thereby enhancing the outcomes of PDT 201. Integrating NGs with photothermal capabilities holds promise for synergistic effects with PDT. Another strategy involves utilizing a tumor-specific nanogenerator that generates peroxynitrite to enhance vascular permeability and oxygen supply within the TME 22. The availability of blood supply to organs not only impacts the absorption and distribution of PSs but also plays a role in influencing the overall effectiveness of PDT. The proportions of PSs binding to tumor tissue after transport through the bloodstream contribute to these variations. In a related development, Jie *et al.* developed an integrated device based on the triboelectric effect for detecting dopamine in alkaline conditions^34^. Dopamine has been shown to enhance the efficacy of anticancer drugs and may act as an antiangiogenic agent in the treatment of breast and colon cancer 154. Combining this innovative device with TENG holds significant potential for accurately determining dopamine levels in cancer treatment settings.

PDT exhibits a dual nature in its potential to address tumor metastasis. Firstly, PDT exhibits the ability to trigger immune responses that can impede metastasis 202. These responses hold promise for sustained protection against metastatic dissemination 31, 32, 203. However, translating this potential into clinical effectiveness encounters obstacles due to the intricacies of metastatic pathways, tumor diversity, and the necessity for refined treatment regimens 93, 204. Murillo *et al.* demonstrated a ZnO nanosheets based PENG to stimulate macrophage motility 20, suggests a potential strategy to enhance PDT's ability to activate immune responses. The enhanced mobility of macrophages could potentially enhance immune cell recruitment and activation at the tumor site post-PDT. Further research is imperative to explore the synergistic effects of combining PENG-induced macrophage stimulation with PDT to enhance immune response, presenting a novel avenue to bolster PDT efficacy against tumor metastasis. Moreover, strategies are essential to reduce CTCs and monitor immune status promptly. Nanotechnology enhances cancer cell adhesion, CTC analysis, and immune status detection 19, 165. These benefits facilitate accurate tracking of tumor metastasis and immune status, guiding the integration of PDT with other therapies to effectively reduce tumor recurrence and metastasis.

## 5. Overcoming Tumor Resistance to PDT at the Subcellular Level

### 5.1. NGs for Modulating PDT-induced Cell Death

Apoptosis, primarily triggered by ROS-mediated oxidative stress during PDT, represents the main mechanism of cell death induced by PDT 205. Excessive ROS production during photodynamic reactions leads to structural and functional changes in cancer cells 206, activating caspase proteins that initiate signal transduction leading to apoptosis 207. In contrast, necrosis refers to cell injury that causes premature cell death through autolysis 208. Shifting the mode of cell death induced by PDT from apoptosis to necrosis can intensify the damage inflicted on cancer cells 205. Necrosis can occur through various mechanisms, such as the direct targeting of the cell membrane by PSs, ROS-induced activation of necrotic pathways, ATP depletion leading to cell death, or inhibition of the autophagy pathway. Several factors influence necrotic cell death in cancer cells exposed to PDT, including the subcellular localization of PSs, ATP depletion in the cytosol 209, and the specific type of ROS generated during the process 210.

High doses of PDT have the potential to shift the mode of cell death from apoptosis to necrosis by increasing levels of ROS 211. NGs can enhance ROS production, while ATP depletion can further promote necrosis. In a study conducted by Yang and colleagues 26, a TENG-supported device generated higher levels of ROS, including superoxide anion (O_2_^-^•), hydroperoxide radical (HOO•), peroxides (H_2_O_2_, ROOH), and hydroxyl radical (HO•), initiating free radical chain reactions that induce various cell death pathways, including apoptosis, necrosis, and other cell death mechanisms.

Additionally, Xue *et al.* proposed a high-performance TENG-based glucose sensor capable of detecting glucose concentrations without the need for glucose enzymes 153. This device has the potential to monitor glucose levels, which can be instrumental in assessing the status of cellular ATP levels. Considering the differential immune responses elicited by necrosis and apoptosis, combining these strategies with PDT-induced anti-tumor immunity shows promise and merits further investigation.

In advanced tumors, serves as a mechanism activated to support cell survival and provide energy acquisition 212. Cancer cells that are resistant to PDT often exhibit elevated levels of the anti-apoptotic protein Bcl-2, which functions to shield them from PDT-induced phototoxicity 212. Studies have shown that the efficacy of PDT can be improved in cells deficient in the autophagy-related protein5 (ATG5), such as in HeLa and MCF-7 cells, indicating the involvement of autophagy in the adaptive survival response of cells undergoing PDT treatment 213. To address the challenge of cell survival in PDT-resistant cancer cells, a self-powered TENG has been developed to deliver siRNA targeting genes such as Bcl-2, ATG5 and others to overcome cell survival and proliferation in PDT-resistant cancer cells 33.

The impact of autophagy on cell fate largely depends on the dosage of PDT and ROS generated. At lower doses, autophagy can promote cell survival by recycling damaged organelles. However, at higher doses, autophagy may contribute to organelle damage and ultimately lead to cell death. Research has demonstrated that the TENG-Cat System can enhance ROS production, 33, preventing damaged cells from recycling their impaired organelles and cytoplasmic components 214. This disruption in the autophagic process may render cells less resistant to PDT-induced damage, potentially improving the effectiveness of PDT in treating resistant cancer cells.

### 5.2. NGs for Pro-survival Signaling Pathways

PDT can induce various forms of cell death, including apoptosis, necrosis, or autophagic cell death in tumors. However, cancer cells often activate pro-survival signaling pathways such as Bcl-2, HIF-1, AMPK, NRF2, NF-kB, and AKT-TOR to adapt and survive the treatment, which can lead to PDT resistance 215. Inhibiting these pathways has the potential to enhance the efficacy of PDT.

An integrated TENG system has been developed to efficiently delivers siRNA targeting Bcl-2, HIF1-α, or NF-κB to overcome resistance to PDT 207. The AMPK signaling pathway, which is involved in regulating autophagy, plays a role in contributing to PDT resistance 213, 216. For example, the activation of autophagy through the AMPK pathway induced by 5-aminolevulinic acid (5-ALA) photoactivation can lead to tumor resistance and reduced apoptosis 125.

NGs have shown effectiveness in modulating cell activities 217-224. PENGs have been utilized to modulate signaling pathways like MAPK/ERK and cAMP to promote differentiation in specific cell types, such as PC12 cells 18. Furthermore, electrical stimulation has been shown to activate AMPK, thereby suppressing autophagy and mTOR-HIF-1 signaling pathways. This suppression can enhance apoptosis, potentially overcoming drug resistance in cancer cells. By targeting these signaling pathways and utilizing innovative nanogenerator systems, researchers aim to improve the effectiveness of PDT and combat resistance mechanisms in cancer treatment.

### 5.3. NGs for the PSs Excretion and Endocytosis

Intracellular accumulation of PSs is crucial for PDT's cytotoxic effects on tumor cells. However, resistance to PDT can arise due to the excretion of PSs by multidrug resistance transporters such as P-glycoprotein (P-GP) and Breast cancer resistance protein (BCRP/ABCG-2) 117. Blocking P-GP with Verapamil or ABCG-2 with tyrosine kinase inhibitors like Imatinib mesylate can increase the intracellular content of PSs, thereby enhancing the effectiveness of PDT 225 226. Overexpression of P-GP and ABCG-2 can reduce the intracellular concentration of PSs, impacting the photosensitivity of cells 226. NGs have been shown to down-regulate P-GP and ABCG-2, offering a potential strategy to overcome PDT resistance mediated by these transporters 33.

Mitochondrial dysfunction and impaired ATP synthesis can reduce the drug transport capacity of P-GP in cancer cells. A high-performance MXene/GO-based TENG serves as a noninvasive glucose sensor capable of detecting glucose levels without the need for a glucose enzyme 153. Monitoring glucose levels is crucial as glucose is metabolized to ATP, which supports the function of the P-GP efflux pump. By detecting glucose concentration, the MXene/GO-based TENG for noninvasive glucose detection may provide insights into monitoring the function of the P-GP efflux pump to some extent. This innovative approach could potentially aid in understanding and optimizing the intracellular accumulation of PSs in tumor cells, thereby overcoming resistance mechanisms and enhancing the efficacy of PDT in cancer treatment.

In the realm of PDT, the selective accumulation of PSs in diseased targets is critical for enhancing treatment outcomes and minimizing non-specific damage to healthy tissues 227. Endocytosis is the initial step in PS absorption, facilitating their accumulation within cells 228. However, the selective endocytosis of PSs in cancer cells is often hindered by the hydrophobicity of many PS compounds 118. Even for hydrophilic PSs, the selective accumulation in diseased targets may not be sufficient for clinical use 229. To address these challenges, various nanoparticle-based delivery systems, such as liposomes, polymeric micelles, and silica-based nanoparticles, have been developed to improve the selective accumulation of PSs 230-234. While these methods have shown some benefits, they do not provide comprehensive solutions, as issues remain in terms of sustained delivery, the delivery of large molecular weight PSs, and the targeting of deep-seated or irregularly shaped tumors.

Recent studies have explored alternative strategies to enhance PS delivery and endocytosis. For instance, Yang *et al.* demonstrated efficient delivery of exogenous materials, including large molecules, into hard-to-transfect primary cells with high cell viability 33. Additionally, a TENG-powered electrochemotherapy system with microneedle electrodes showed promising results for 3D cell or tissue models, enhancing drug delivery in irregularly shaped tumors 24. Furthermore, a self-powered drug delivery system comprising a current source derived from a disk TENG (D-TENG) enabled sustained drug delivery and significant enhancement of cancer cell uptake over long-term conditions 235. Incorporating PSs into such self-powered electrical systems utilizing TENGs has demonstrated the potential to promote endocytosis and increase PS accumulation within tumor cells^23^. By capitalizing on these innovative delivery strategies and self-powered electrical systems, the selective accumulation of PSs in diseased targets can be further enhanced, ultimately improving the efficacy and clinical applicability of PDT.

### 5.4. NGs for Gene Therapy

NGs have been shown to increase plasma membrane potential and permeability, thereby enabling efficient delivery of siRNA and transfection of DNA plasmids into cells. This approach offers several advantages over conventional methods like electroporation, including high delivery efficiency and improved cell viability.

The self-regulated electro system driven by TENG/PENG has proven effective for gene transfection and siRNA delivery in cancer treatment 33. An integrated system developed by Zhao *et al.* utilizes a self-powered TENG to provide a stable voltage pulse source, triggering an increase in plasma membrane potential and permeability 24. This system achieves efficient delivery of various molecules into different cell types, with delivery efficiency reaching up to 90% and cell viability exceeding 94%. This device can facilitate siRNA delivery for PDT, enabling gene therapy to overcome drug resistance by silencing genes involved in PDT resistance.

Additionally, a fully integrated electrical stimulation cell culture dish (SESD) has been designed to provide self-powered electrical stimulation to adherent cells 236. Electrical stimulation safely increases intracellular calcium concentration by opening calcium-ion channels, significantly enhancing plasmid transfection efficiency in mammalian cells and promoting cell survival.

Another self-powered electroporation system, utilizing a polypyrrole microfoam electrode, has been developed to deliver biomolecules with high efficiency and cell viability 237. By leveraging these technologies, the integrated system based on NGs can efficiently deliver siRNA and enhance DNA plasmid transfection, enabling the modulation of tumor resistance in PDT. This innovative approach holds promise for improving gene delivery and therapeutic outcomes in cancer treatment.

**Figure 5:**
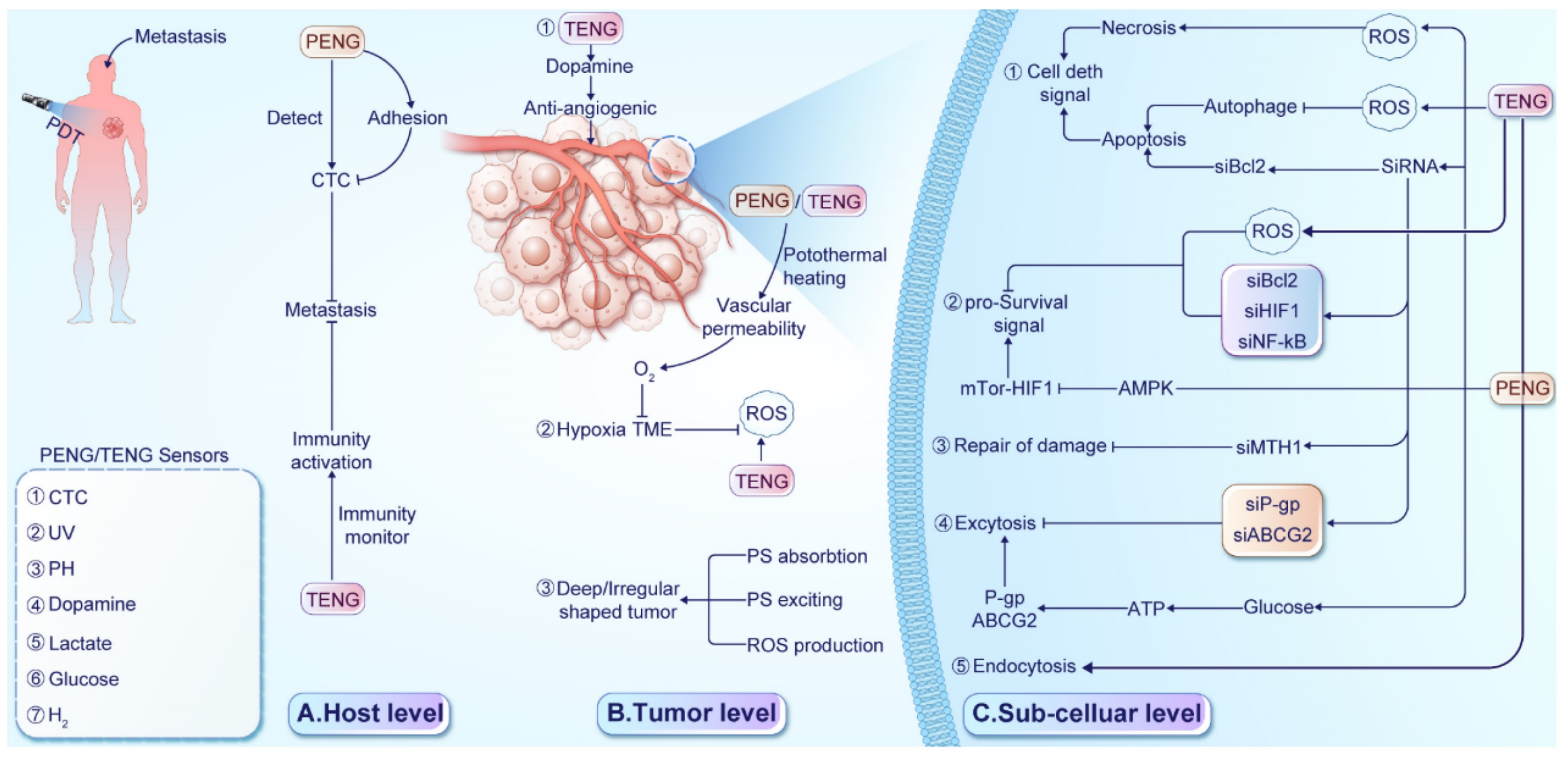
Strategies employed by NGs to overcome tumor resistance to PDT. (A) At the host level, NG-supported devices detect various biomarkers for early identification of metastasis and enhance immune activity to improve survival. (B) At the tumor level, ①A TENG-based sensor detect dopamine levels with anti-angiogenic properties. ② PENG/TENG systems facilitate thermal therapy, increasing O2 supply, relieving hypoxia, and enhancing ROS production. RR-TENG or TENG-Cat directly stimulate ROS production. ③ PENG/TENG-supported drug delivery systems(DDSs) enhance PS absorption, excitation, and ROS production for deep-seated or irregularly shaped tumors. (C) At the subcellular level, ① NG devices influence cell death signals, with TENG enhancing ROS production and inducing necrosis or enhancing apoptosis through Bcl-2 gene silencing or autophagy suppression. ② NG devices suppress pro-survival signals by increasing ROS production and silencing pro-survival genes or activating the AMPK signal to regulate mTOR-HIF1 signaling. ③Silencing the Mutt homolog 1(MTH1) gene reduces protein repair damaged by ROS. ④ TENG devices silence P-gp and ABCG2 to reduce photosensitizer exocytosis. TENG-based glucose sensors detect ATP production. ⑤ A designed D-TENG enhances cancer cell endocytosis and PS absorption.

Figure 5 presents a comprehensive survey of strategies employing NGs to combat barriers in PDT. NGs exhibit beneficial characteristics, including precise and effective delivery of PSs, ROS generation under hypoxic conditions, and facilitation of biomedical sensors for detecting PDT byproducts and materials that influence treatment outcomes. Additionally, NGs promote the permeability and intracellular penetration of therapeutic agents like PSs, small interfering RNA (siRNA), and DNA plasmids. The programmable nature of NGs allows for the fine-tuning of photodynamic and physiological processes within tumors and the human body, ultimately optimizing PDT efficacy.

## 6. Conclusion

In this study, we have investigated the capacity of Nanogenerators (NGs) to surmount resistance in PDT, presenting novel approaches to bolster treatment effectiveness. PDT resistance poses a notable obstacle, resulting in subpar results in specific scenarios. Nevertheless, the amalgamation of NGs with PDT exhibits substantial potential in tackling this hurdle. NGs present distinctive benefits by furnishing a localized and sustainable energy reservoir for PDT, thereby circumventing drawbacks linked to conventional light sources. NGs can convert mechanical, frictional, or thermal energy into electrical energy to power the light source for PS activation and ROS generation.

We have explored various types of NGs, encompassing nanowires, nanofibers, and 2D material-based NGs, emphasizing their potential roles in PDT. Integration of these NGs with photosensitizers holds promise for enhancing delivery efficiency, augmenting cellular uptake, and enabling controlled release, thereby heightening the therapeutic efficacy of PDT. Furthermore, NGs offer a means to counter resistance mechanisms commonly encountered in PDT, including hypoxia, limited tissue penetration, and multidrug resistance. By furnishing an oxygen-independent energy source, NGs effectively tackle challenges posed by hypoxic tumor microenvironments. Their unique attributes, such as small size and flexibility, facilitate deep tissue penetration and targeted delivery, enabling effective treatment of tumors in anatomically intricate locations. Moreover, NGs possess the potential to surmount multidrug resistance by triggering cell death pathways that operate independently of drug efflux pumps or cellular resistance mechanisms. This breakthrough opens new avenues for combating drug-resistant tumors, broadening the therapeutic repertoire accessible to medical practitioners.

While NG-PDT technology shows great promise, critical challenges must be tackled to enhance effectiveness and practicality. These include ensuring biocompatibility to prevent adverse immune reactions, developing scalable manufacturing processes for mass production, improving targeting precision to deliver PSs accurately, obtaining regulatory approval for clinical integration, ensuring long-term stability, addressing emerging resistance mechanisms, exploring combination therapies, optimizing cost-effectiveness, and integrating imaging and monitoring for personalized treatment planning. Through collaborative research and innovative approaches, overcoming these challenges can propel the progress of NG-PDT technology, enhancing its efficacy and clinical relevance in cancer therapy.

The development of NG-based PDT technology presents several promising opportunities for advancement. Leveraging NGs, researchers can enable personalized treatment approaches by tailoring PDT regimens to individual patient and cancer characteristics. Multifunctional NG platforms integrating PDT with imaging, drug delivery, or other therapeutic modalities can enhance treatment efficacy and precision. Smart NG-based drug delivery systems allow for on-demand photosensitizer release in response to specific tumor microenvironment stimuli. Theranostic NG applications integrate therapeutic and diagnostic capabilities, enabling real-time monitoring of treatment efficacy. Improved NG targeting can selectively deliver PSs to cancer cells, minimizing off-target effects. Synergistic combinations of NG-PDT with immunotherapy or targeted therapy show potential to overcome resistance mechanisms and achieve enhanced therapeutic outcomes. Remote monitoring and control of PDT parameters through NG systems can offer greater flexibility and precision in treatment administration. Integrating bioinformatics and AI algorithms can optimize NG-PDT protocols based on individual patient profiles. NG-based strategies to mitigate therapeutic resistance in cancer cells can improve long-term treatment responses. Promoting global accessibility of NG-PDT technology through optimized manufacturing and cost-reduction measures can expand its clinical applicability. By capitalizing on these opportunities through collaborative research efforts, the development of NG-PDT technology can be advanced, leading to improved cancer treatment outcomes.

In conclusion, NGs present a promising avenue for overcoming resistance in PDT through the provision of localized and sustainable energy sources, ultimately enhancing treatment effectiveness. The fusion of NGs with PDT stands to transform cancer therapy and elevate patient outcomes. Ongoing exploration and advancement in this realm are poised to drive the clinical implementation of NG-PDT methodologies, offering benefits to patients globally and propelling advancements in the domain of cancer therapeutics.

## Abbreviations

2-DG: 2-deoxy-D-glucose
3D: three dimensional
5-ALA: 5-aminolevulinic acid
ABCG2: ATP-binding cassette transporter G2
AKT: Protein kinase B
AMPK: AMP-activated kinase
ATG5: autophogy-related 5
ATP: Adenosine Triphosphate
BBB: blood-brain barrier
BCRP: Breast Cancer Resistance Protein
cAMP: cyclic adenosine monophosphate
CLW: composite linear worm
COF-CNT: covalent organic framework on a carbon nanotube
CTCs: circulating tumor cells
CWPW: composite wavy pattern worm
DDS: drug delivery system
DNA: deoxyribonucleic acid
EF: electrical field
ER: endoplasmic reticulum
ERK: extracellular signal-regulated kinases
FIN: ferroptosis inducer
GaN: gallium nitride
GOx: Glucose oxidase
GSH: glutathione
GST: glutathione S-transferase
GPx4: glutathione peroxidase 4
HIF-1: hypoxia-inducible factor 1
HO•: hydroxyl radical
HOO•: hydroperoxide radical
IKE: imidazole ketone erastin
iNOS/NOS2: inducible nitric oxide synthase
LED: light emitting diode
LIP: labile iron pool
MAPK: mitogen-activated protein kinases
MDR: multidrug resistance
MDSCs: myeloid-derived suppressor cells
MSM: metal-semiconductor-metal
MTH1: Mutt homolog 1
mTOR: Mammalian target of rapamycin
NE: nanotechnology-enabled
NF-κB: Nuclear factor-kappaB
NGs: nanogenerators
NIR: near-infrared
NMOF: Titanium-Based Nanoscale Metal-Organic Framework
NO: nitric oxide
NOS: nitric oxide synthase
O_2_•-: superoxide anions
O_2_^-^: superoxide radicals
OH•: hydroxyl radicals
OXPHOS: oxidative phosphorylation
PDMS: polydimethylsiloxane
PDT: photodynamic therapy
PENGs: Piezoelectric Nanogenerators
PET: polyethylene terephthalate
P-gp: P-glycoprotein
MDR: multidrug resistance protein
PMU: power management unit
PSs: photosensitizers
ROS: reactive oxygen species
SDT: sonodynamic hybrid therapy
siRNA: small interfering RNA
SOD: superoxide dismutase
TENGs: Triboelectric Nanogenerators
TME: tumor microenvironment
UV-Vis: ultraviolet-visible
